# Advanced Biomaterials for Food Edible Coatings

**DOI:** 10.3390/ijms24129929

**Published:** 2023-06-09

**Authors:** C. Valeria L. Giosafatto, Raffaele Porta

**Affiliations:** Department of Chemical Sciences, University of Naples Federico II, Via Cynthia, 80126 Naples, Italy; giosafat@unina.it

The aim of this Special Issue is to highlight recent investigations on different biopolymers obtained from renewable sources for use as edible coatings. In particular, the most rapidly expanding field is the tuning of innovative packaging materials for the food industries, especially in light of growing environmental and health concerns. In the last ten years there has indeed been an increasing effort to obtain sustainable materials to exploit as packaging [1], mostly represented by films formulated from biodegradable and/or edible components.

In this respect, various biomacromolecules of vegetal and animal origin, such as homo- and heteropolysaccharides or proteins, were utilized for the manufacture of novel coatings. These biopolymers were sometimes further modified by functionalization with diverse molecules to improve the technological and biological features of the produced materials (Figure 1). Achieving high-quality coated food products is mainly dependent on the characteristics of the specific food to be coated, the properties of the components of the coating solution, and the prepared coating material.

The contributions to the present Special Issue consist of two reviews and eight scientific articles. The first review describes the possible use of nanosystems as edible coatings for food preservation [2] and, in particular, the recent developments in the use of nanoparticles, nanotubes, nanocomposites, and nanoemulsions are discussed critically. The second review describes probiotic incorporation into edible films and coatings as a bioactive solution to produce functional foods, providing significant information regarding probiotics and bioactive packaging and their applications, as well as discussing certain limitations of their use, the current legislation, and the future trends [3].

Several of the scientific articles detail studies on the development of polysaccharide-derived films. Senturk Parreidt et al. [4] investigated different concentrations of sodium alginate as a film matrix component in the presence of plasticizers (glycerol or sorbitol), different surfactants (tween 40 and 80, span 60 and 80, and lecithin), and vegetable oils (sunflower, olive, and rapeseed oils). Different combinations of the component were formulated, and the derived coatings were analyzed. Three formulations were designed, and their stability and wettability tests on strawberry showed that they could be successfully used in coating applications. Bekhit et al. [5] described novel bioactive films developed by incorporation of *Lactococcus lactis,* free or previously encapsulated in alginate–pectin composite hydrogel microbead, into cellulose derivative (hydroxylpropylmethylcellulose (HPMC) and corn starch films. The authors showed that the polysaccharide-based films containing encapsulated *Lactococcus lactis* showed a complete inhibition of Listeria growth during the first five days of storage at 5 °C and a reduction of 5 logs after 12 days.

Sagnelli et al. [6] investigated another polysaccharide-based film consisting of amylose-only (AO) starch obtained from transgenic lines of barley. This kind of starch was generated by RNA interference against all three starch branching enzymes in barley [7]. The AO starch was shown to provide a useful raw material for bio-plastic fabrication as it possesses an improved film-forming ability due to its increased amylose content. In another study, Cao and coworkers [8] utilized inulin combined with chitosan to prepare composite films, together with oregano and thyme essential oils (EOs) to confer the films with bioactivities, and suggested that these films could be used as an active packaging material in the food industry. Conversely, the aim of the study of Escamilla-Garcia et al. [9] was to develop edible films based on a chitosan–zein mixture with three different EOs added: anise, orange, and cinnamon. The EO-containing films were shown to be able to inhibit the growth of *Penicillium* sp. and *Rhizopus* sp. to a larger extent than those prepared without EOs, probably due to the structural changes of the film matrix caused by the chemical interactions among amino acid side chains from zein, glucosamine from chitosan, and cinnamaldehyde, anethole, or limonene from the EOs.

Furthermore, two additional articles highlighted the role that both gums and emulsifiers play in preserving food quality and ensuring food safety. In this respect, Jia et al. [10] investigated the effects of a guar gum and sorbitol coating on the oil absorption of French fries. The reported results showed that pretreatment by blanching with calcium ions and coating with guar gum and sorbitol significantly reduced the structural oil and penetrated surface oil of French fries, with no negative effects on food texture. On the other hand, Arredondo-Ochoa et al. [11] studied the effects of various emulsifiers (stearic acid, Tween 80, or Tween 80/Span 60) as well as of different emulsification processes (homogenization, ultrasound, or microfluidization) on formation of nanoemulsions based on oxidized corn starch, beeswax, and natural antimicrobials, such as lauric arginate and natamycin. Regardless of emulsifier type and emulsification process, the authors demonstrated that beeswax–starch nanoemulsions applied on tomato surfaces exhibited low contact angles and effective wettability, thus suggesting their use as edible coatings to preserve the quality and safety of fresh products.

Finally, the last few decades have seen an increase in interest in protein-based coating materials because of their potential to protect pharmaceuticals or extend the shelf life of different food products [12]. As with other biopolymers, proteins also need to be mixed with different types of plasticizers to increase film extensibility, to reduce its crystallinity, as well as to improve its vapor barrier properties [13]. Besides the known polyols, such as glycerol and sorbitol, the effect of the aliphatic polyamines spermidine and spermine on both polysaccharide- and protein-based films was recently studied [14,15]. The last article of this Special Issue [16] focused on films obtained from proteins extracted from bitter vetch (*Vicia ervilia*), an annual legume species of the *Vicia* genus that is, to date, widely cultivated only for animal feed in temperate regions of Europe, western and central Asia, north Africa, and the Americas. Bitter vetch seeds, containing up to 25% protein, were demonstrated to be a potentially useful source for food packaging applications, being not only an abundant protein source but also an inexpensive one [17,18]. In the reported article, the authors demonstrated that spermidine not only is able to act as a plasticizer itself by ionically interacting with proteins, but that it also facilitates glycerol-dependent reduction of the intermolecular forces along the protein chains, consequently improving film flexibility and extensibility. Thus, spermidine may be considered not only as a primary, but also as a secondary, plasticizer because of its ability to enhance glycerol plasticizing performance.

In conclusion, it is sincerely hoped that the scientific reports published in the present Special Issue will be successful in providing new research insights for the manufacture of innovative bio-based edible coatings to replace the petroleum-based plastics that are harmful for the entire ecosystem. Biodegradable active packaging, i.e., materials able to promote food preservation while avoiding plastic waste accumulation, as a new generation of food packaging may be able to play a key role in this context. Materials derived from bio-based polymers, like polysaccharides and proteins, have emerged as renewable resources and might be leading actors in the redesigning of plastic materials under a “green perspective”.

## Figures and Tables

**Figure 1:**
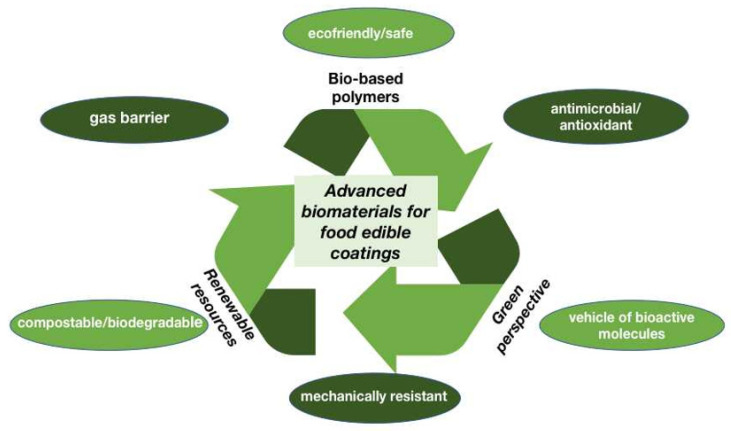
Advanced biomaterials obtained from renewable resources for sustainable food applications.
